# How spectroscopy and microspectroscopy of degraded wood contribute to understand fungal wood decay

**DOI:** 10.1007/s00253-012-4369-5

**Published:** 2012-09-16

**Authors:** Karin Fackler, Manfred Schwanninger

**Affiliations:** 1 Institute of Chemical Engineering, Vienna University of Technology, Gumpendorfer Straße 1a, 1060 Vienna, Austria; 2 Department of Chemistry, BOKU - University of Natural Resources and Life Sciences, Vienna, Austria

**Keywords:** White-rot fungi, Brown-rot fungi, Wood degradation, Nuclear magnetic resonance spectroscopy, NMR spectroscopy, Near infrared spectroscopy, Mid infrared spectroscopy, NIR spectroscopy, MIR spectroscopy, UV spectroscopy, Microspectroscopy, Imaging

## Abstract

Nuclear magnetic resonance, mid and near infrared, and ultra violet (UV) spectra of wood contain information on its chemistry and composition. When solid wood samples are analysed, information on the molecular structure of the lignocellulose complex of wood e.g. crystallinity of polysaccharides and the orientation of the polymers in wood cell walls can also be gained. UV and infrared spectroscopy allow also for spatially resolved spectroscopy, and state-of-the-art mapping and imaging systems have been able to provide local information on wood chemistry and structure at the level of wood cells (with IR) or cell wall layers (with UV). During the last decades, these methods have also proven useful to follow alterations of the composition, chemistry and physics of the substrate wood after fungi had grown on it as well as changes of the interactions between the wood polymers within the lignocellulose complex caused by decay fungi. This review provides an overview on how molecular spectroscopic methods could contribute to understand these degradation processes and were able to characterise and localise fungal wood decay in its various stages starting from the incipient and early ones even if the major share of research focussed on advanced decay. Practical issues such as requirements in terms of sample preparation and sample form and present examples of optimised data analysis will also be addressed to be able to detect and characterise the generally highly variable microbial degradation processes within their highly variable substrate wood.

## Introduction

The main structural wood polymers—cellulose, hemicelluloses and lignin—are the most abundant biopolymers in the Earth's carbon cycle. These polymers form the lignocellulose complex in all woody tissues. Its highly ordered structure of cellulose microfibril aggregates embedded in a matrix of hemicelluloses and lignin provides the basis for its mechanical strength (Salmén and Burgert 2009) and for the resistance to microbial attack (Daniel 2003), to which also low molecular mass extractives contribute (Zabel and Morrell 1992).

Wood therefore is a remarkably durable material. In nature, only higher fungi have developed biochemical systems to degrade the lignocellulose complex and perform the conversion and mineralisation of wood to carbon dioxide and water. Most of these fungi belong to the Basidiomycetes. Although they are phylogenetically closely related (Binder and Hibbett 2002; Floudas et al. 2012) their strategies of degrading wood are diverse: While brown-rot fungi degrade primarily the wood polysaccharides and leave behind a polymeric but highly modified lignin, simultaneous white-rot fungi degrade all polymeric wood constituents at similar rates. Selective white-rot fungi, which lack the ability to degrade cellulose efficiently, cause extensive delignification of wood. Ascomycetes and Deuteromycetes may cause soft-rot decay that leads to softening of wet wood. Cavity formations in wood cell walls are most characteristic for this decay type. Extensive reviews on decay pattern, chemistry, and biochemistry of microbial wood degradation are available (Daniel 2003; Eriksson et al. 1990; Goodell 2003).

The natural processes occurring during fungal wood degradation may be utilised for industrial purposes and have a great potential for cellulose-producing and wood-processing industries as well as for high value-added conversion of lignocellulosic waste materials in Biorefineries. Particularly, the molecular mechanisms of selective white-rot fungi offer a series of applications in the field of biotechnology of renewable resources (Bajpai 2012).

However, Basidiomycetes are not only microorganisms of industrial interest. They are also considered as pest organisms on construction wood, and it is of particular importance to detect wood decay by sensitive methods as early as possible. Although the mechanical properties of wood suffer quite early in the decay (Winandy and Morrell 1993), it is difficult to trace the chemical alterations related to the mechanical ones because they are too small to be detected with conventional and standardised wet-laboratory methods. Moreover, the information related to molecular structure i.e. the interactions between and within the macromolecules of the lignocellulose complex is lost when samples are analysed with degradative chemical methods, and separation or isolation methods may lead to non-representative results.

Molecular spectroscopy—ultra violet (UV), near infrared (NIR) and mid infrared (MIR) spectroscopy, and nuclear magnetic resonance (NMR) spectroscopy provide less invasive or even non-destructive methods that have been proven well suited to investigate the chemical composition as well as the molecular structure of wood and functional groups of its constituents. It is therefore the aim of this review to present an overview on how these methods could contribute to understand the action of wood degrading fungi on their actual substrate wood and what kind of chemical and microstructural alterations take place during decay. In addition, UV and IR microspectroscopy provide the opportunity to localise these alterations within the cellular and chemically and structurally highly variable substrate wood. In this review, mainly original research publications were considered which focussed on spectroscopic analyses of fungal wood degradation without applying means of chemical degradation and separation.

## Potentials of spectroscopic methods to analyse fungal wood degradation processes

The (molecular) spectroscopic methods used in the context of fungal wood degradation were NMR, NIR and MIR spectroscopy and Fourier transform (FT)-IR and UV microspectroscopy. Although Raman spectroscopy would perfectly fit into the scope of this review, we were not able to find peer-reviewed literature on that topic.

The methods and their potential applications for wood and wood processing have been reviewed elsewhere (Gil and Pascoal Neto 1999; Maunu 2002; Moore and Owen 2001; Tsuchikawa 2007; Tsuchikawa and Schwanninger 2011). Thus, this review focus on practical issues such as sample form and preparation (Table 1) and intends to provide insights into the information that can be potentially gained from the spectral data also with the aid of advanced data analysis tools.

**Table 1:** Spectroscopy techniques

	Wavelength	Spatial resolution in microspectroscopy	Typical sample form	Information related to wood degradation	Remarks
NMR	Several metres	n.a.	Solid wood, Dissolved wood components e.g. lignin	Composition, Functional groups, Molecular structure, Mobility of molecules	
MIR	2.5–25 μm	2.5–14 μm^a^; 2.8–12.5 μm^b^; typically 2–6.25 μm pixel size	wood thin sections (~ 10 μm), wood surfaces, milled wood, powders	Composition, Functional groups, Molecular structure	Water bands overlap with wood related bands
NIR	0.8–2.5 μm	1.0–2.5 μm	wood thin sections (~100 μm), wood surfaces, milled wood, powders	Composition, C–H, O–H groups, Molecular structure	Bands of adsorbed water overlap with wood related bands
UV	Typically 280 nm	250 nm pixel size	Epoxy resin embedded wood thin sections (0.5–1 μm)	Lignin content	

^a^With mercury cadmium telluride (MCT) detectors

^b^With focal plane array imaging detectors

### Nuclear magnetic resonance spectroscopy

Solid state NMR (^13^C cross polarisation magic angle spinning nuclear magnetic resonance spectroscopy, ^13^C CPMAS NMR) is a technique to analyse solid (wood) samples. The contents of the main wood components (cellulose, hemicelluloses, lignin and extractives) and their molecular structure can be assessed, because chemical shifts in ^13^C CPMAS NMR spectra are related to the molecular environment of carbon atoms within the sample. Chemical shifts for functional groups of lignin and carbohydrates have been reviewed and summarised by Gil and Pascoal Neto (1999).

It was NMR spectroscopy that allowed Atalla and Vanderhart (1984) to discover two crystal allomorphs in native cellulose I—cellulose I_α_ and cellulose I_β_. Furthermore, through spectral fitting of the C4 cellulose region, the quantification of the various cellulose structural features within wood cell walls and fibres is possible (Larsson et al. 1997; Wickholm et al. 1998). Thus, cellulose crystallinity and the contents of cellulose crystal allomorphs as well as hemicelluloses content can be evaluated from ^13^C CPMAS NMR spectra.

Low field NMR (synonyms: time resolved NMR, wide-line solid state NMR) can be applied to measure the mobility of molecules within wood cell walls (Gilardi et al. 1994).

Lu and Ralph (2003) developed a preparation method for analysing wood with solution state NMR. After extensive disintegration by ball-milling, wood can be dissolved in various solvents such as dimethyl sulfoxide and readily be acetylated in this solution. Although structural information related to polysaccharides is lost during this treatment, the method allows for representative analysis of lignin structures using ^1^ H–^13^C heteronuclear single quantum correlation (HSQC).

### Mid infrared spectroscopy (MIR or IR or FT-IR)

IR is now a fast and convenient spectroscopic method applicable for the analysis of solid wood samples requiring a minimum of sample preparation. During recent years, also various technical solutions for spatially resolved spectroscopy became available—microspectroscopy and IR imaging microscopy. With these methods, chemical and microstructural information on the cellular and to some extent also on the subcellular level can be gained. Absorption bands in the MIR region of the electromagnetic spectrum derive from the excitation of fundamental molecular vibrations of chemical bonds. Vibration frequencies depend on the strength of the chemical bonds and on the masses of the involved atoms. Comprehensive assignments of infrared bands for wood are found in the literature (Faix 1991; Liang and Marchessault 1959a, b; Schwanninger et al. 2004b).

Information related to the molecular structure of the wood polymers are mainly found in the spectral region of O–H vibrations (Maréchal and Chanzy 2000; Niduszynski and Marchessault 1972) but also in the so-called fingerprint region where bands can be related to cellulose crystallinity (Barsberg 2010; Kataoka and Kondo 1998; O'Connor et al. 1958; Richter 1991; Richter et al. 1991) and to cellulose I crystal allomorphs (Åkerholm et al. 2004).

From the practical point of view, highest signal to noise ratios can be acquired in transmission mode using the potassium bromide (KBr) technique, where 1–2 mg of milled wood are diluted and homogenised in about 200–300 mg of KBr and pressed to a dense pellet. Alternatively, very thin sections of wood of 10–30 μm thickness can be measured in transmission mode. The latter sample preparation is also appropriate for transmission FT-IR microspectroscopy or FT-IR mapping or imaging microspectroscopy (Fackler et al. 2010).

More convenient in terms of sample preparation is a method, where IR radiation (diffusely) reflected from the sample surface is detected (diffuse reflectance infrared Fourier transform (DRIFT) spectroscopy). However, dilution of a milled sample with KBr is also advantageous in this technique (Faix and Böttcher 1992; Ferraz et al. 2000). Nowadays, however, spectra from wood surfaces and spectra of milled wood are mostly acquired applying the (attenuated total reflection (ATR)-FT-IR technique, where the sample is pressed onto a crystal of high refractive index and the so-called evanescent wave contains the spectral information of the sample surface. ATR-FT-IR spectra can be acquired within seconds.

FT-IR microspectroscopic spectra can be recorded either in transmission, reflection, or with the ATR-technique typically using a Germanium crystal. With the most sensitive detectors of advanced equipment, spectra of high signal to noise ratio can be readily obtained even with a spatial resolution at the diffraction limit. Current state of the art are pixel sizes as small as several square micrometre (Griffiths 2009). Array detectors such as focal plane array detectors or linear array detectors allow for recording up to 16,384 (i.e. 128 × 128) spectra simultaneously. In this way, chemical images of wood surfaces and thin sections can be obtained within several minutes.

### Near infrared spectroscopy

Near infrared spectroscopy is a rapid method that has been most commonly used for applications related to process control also in wood and cellulose fields (Tsuchikawa 2007; Tsuchikawa and Schwanninger 2012). In addition, NIR spectra may yield a lot of information on the chemical composition and on the molecular structure of wood (Fackler and Schwanninger 2010; Schwanninger et al. 2011; Tsuchikawa and Siesler 2003; Watanabe et al. 2006). In the NIR, absorption bands related to overtones of and combinations of fundamental vibrations in the MIR are found. The overtones of C–H and O–H vibrations have been mostly used for descriptive data analyses. The vibration frequency of O–H overtone vibrations is highly dependent on the presence of hydrogen bonds. Therefore, information related to crystal structure—cellulose crystal allomorphs I_α_, I_β_ have different NIR spectra—and the extent of crystallinity is also present in NIR spectra (Fackler and Schwanninger 2010; Inagaki et al. 2010). In the C–H first overtone region, lignin-derived aromatic C-H groups can be differentiated from aliphatic ones of different sources (Schwanninger et al. 2011).

NIR spectra are mostly acquired from wood surfaces or milled wood in diffuse reflectance mode using fibre optic probes or integrating spheres. However, also transmission spectra of up to several 100-μm-thick thin sections with intact cellular structure can be recorded. Transmission NIR spectra recorded in transversal and longitudinal direction to spruce wood tracheids allow for conclusions on the molecular structure within the orthotropic material wood, because polarised NIR radiation causes selective excitation of oriented molecular vibrations. Therefore, degradation or modification of wood polymers oriented in transversal and longitudinal direction can be assessed with this polarised transmission FT-NIR spectroscopy (Fackler and Schwanninger, 2010). Although the information contained in NIR spectra resembles to a large extent that also found in the MIR, NIR spectroscopy possesses several advantages over MIR: due to low absorption coefficients, bulk or thick samples of intact cellular structure can be measured, O-H groups of no, weak, and strong H-bonding are better separated than in the MIR with the weakly H-bonded ones being more pronounced than the strongly H-bonded ones (Watanabe et al. 2006).

### UV microspectroscopy

UV microspectroscopy has been employed to detect and quantify aromatic structures derived from lignin and aromatic extractives that have an absorption peak near 280 nm or structures with conjugated double bonds. Lignin can be localised within wood cell walls with the diffraction limit of spatial resolution of several 100 nm (~250 nm).

## Ways to extract complex information from complex spectra

As a compound material of the biopolymers cellulose, hemicelluloses and lignin and the presence of low molecular mass organic compounds (extractives), wood is a highly complex material. This complexity is also reflected in the MIR and NIR as well as NMR spectra which are characterised by overlapping bands and signals. Comprehensive analysis of the spectral data, therefore, is a challenge.

Degradative methods intend to separate components or selectively assess them qualitatively and quantitatively. In contrast, data gained from spectroscopic methods contain information of the entire sample in one spectrum. Therefore, it is necessary to literally analyse^1^ the data instead of the sample.

In spectroscopy, the absorption of electromagnetic radiation by chemical groups follows Lambert–Beer’s law, which can be applied in UV microspectroscopy e.g. to quantify lignin in a very thin wood section. Because extinction coefficients depend on lignin chemistry (Fergus and Goring 1970), only semi-quantitative analyses can be attempted for wood. For ^13^C CPMAS NMR, quantitativeness of signals is assumed in some cases rendering information on the composition accessible. Thus, knowledge on the content of one characteristic compound or functional group acquired with reference methods may allow for quantification of another group in the sample. In UV and IR, extinction coefficients are highly dependent on the nature of the chemical group, and in addition, other interactions of electromagnetic radiation with matter such as scattering, diffraction, specular and diffuse reflectance have to be considered for real-life solid wood samples. Furthermore, absorption bands are often highly overlapped. For all these reasons, it is difficult to assess the quantitative information directly, and reference methods are required. IR spectroscopy being an indirect or secondary method requires external calibration that can be performed via univariate regression analysis using band height or area, ratios of bands (Pandey and Pitman 2004, Fackler et al. 2007b) or multivariate regression using several bands (multilinear regression) or all data points of a spectral region (e.g. partial least squares regression) (Ferraz et al. 2000, Schwanninger et al. 2004b).

Nevertheless, IR spectroscopy is very sensitive to qualitative and quantitative differences within and between samples, and knowledge on the absolute amounts is not always important.

Other methods to visualise and accentuate the spectral information are deconvolution of overlapping bands, a method most frequently applied to ^13^C CPMAS NMR spectra (Popescu et al. 2010a), calculation of difference spectra and second derivatives, or two-dimensional correlation spectra, which was in the context of fungal wood degradation applied for NMR spectra (Yelle et al. 2008; Yelle et al. 2011) and for IR spectra (Popescu et al. 2010b).

Multivariate data analysis (MVA) offers great opportunities particularly in IR spectroscopy which as a very rapid method that allows the acquisition of high numbers of spectra within very short time. It is the aim of MVA to identify similarities and dissimilarities among spectra with e.g. principal component analysis (PCA) (Fackler and Schwanninger 2010; Fackler et al. 2007c; Jellison et al. 2002; Schwanninger et al. 2004a) or, as mentioned before, to relate the spectral data to reference data (calibration) for the prediction of the composition of new samples. MVA was also well applicable to analyse IR microscopy images (Fackler et al. 2010, 2011) and analysis of NIR spectra with PCA allowed for conclusions on brown-rot degradation that had not been a priori hypothesised (Fackler and Schwanninger 2010). MVA, however, has been rarely used for the analysis of NMR data in this context (Sivonen et al. 2003).

## Observations of alterations due to fungal wood degradation processes

### Changes of wood composition and its estimation

One aspect of fungal wood degradation is that the chemical composition of wood is altered while fungi are growing on it, because wood constituents are degraded at different rates. Observations concerning these changes due to brown-rot, white-rot and soft-rot degradation are the first ones reported for MIR of degraded wood (Takahashi and Nishimoto 1967). A summary of degradation of a large variety of different wood species by an even larger variety of fungal species are given in Table 2.

**Table 2:** Reports on changes of wood composition due to fungal degradation in chronological order

Fungus(rot)	Wood species (sw/hw)	Method and technique	Reference
*Coriolellus palustris* (br)	*Fagus crenata* (hw)	MIR, KBr	Takahashi and Nishimoto 1967
*Coriolus (Trametes) versicolor* (wr)	*Cryptomeria japonica* (sw)		
*Chaetomium globosum* (sr)			
*Fomitopsis pinicola* (br)	*Betula sp.* (hw)	MIR, KBr	Karklins et al. 1975
*Polystictus versicolor* (wr)	*Abies alba* (sw)	UV microscopy	Bauch et al. 1976
*Fomes annosus* (wr)	*P. sylvestris* (sw)		
*Coniophora cerebella* (br)	*Quercus robur* (hw)		
*Chaetomium globosum* (sr)	*F. sylvatica* (hw)		
*Gloeophyllum trabeum* (br)	*Pinus sp.* (southern yellow pine, sw)	MIR, KBr	Nicholas and Schultz 1987
“Brown-rot”	*Picea abies* (sw)	MIR, KBr	Wienhaus et al. 1989
*Ganoderma australe* (wr, palo podrido)	*Eucryphia cordifoli*a (hw)	^13^C CPMAS NMR	Martinez et al. 1991
*Trametes versicolor* (wr)	*Fagus sylvatica* (hw)		
*Pleurotus eryngii* (wr)			
“Brown-rot”			
*Fomitopsis pinicola* (br)	*P. sylvestris* (sw) + alkaline extraction	MIR, KBr	Körner et al. 1992
“White-rot”	*Betula pubescens* (hw)	DRIFT	Hortling et al. 1992
	*Betula pendula* (hw)	^13^C CPMAS NMR	
*C.* (*T.*) *versicolor* (wr)	*Pinus sylvestris* (sw)	^13^C CPMAS NMR	Gilardi et al. 1995
*C. puteana* (br)	*Fagus sylvatica* (hw)	DRIFT	
*Merulius (Phlebia) tremellosus(a), Poria medula-panis, T. versicolor, Punctularia artropurpurescens, C. subvermispora*	*Eucalyptus globulus* (hw)	DRIFT	Ferraz et al. 2000
*Ganoderma aplanatum* (wr)	*Pinus radiata* (sw)		
*Posia cocos, Laetiporus sulfureus* (br)			
*Fomitopsis pinicola* (br)	*Betula pendula* (hw)	DRIFT	Backa et al. 2001
*Stereum hirsutum* (wr)			
*Ceriporiopsis subvermispora* (wr)	*Pinus radiata* (sw)	DRIFT	Ferraz et al. 2001
*Ganoderma australe* (wr)			
*C. puteana* (br)	*Entandrophragma cylindricum* (hw)	UV microscopy	Kleist and Schmitt 2001
*G. trabeum, P. placenta* (br)	*P. abies* (sw)	NIR (diffuse reflectance)	Kelley et al. 2002
*Coniophora puteana* (br)	*Pinus sylvestris* (sw)	FT-MIR, KBr	Pandey and Pitman 2003
*C. (T.) versicolor* (wr)			
*Phanerochaete chrysosporium* (wr)			
*C. puteana* (br)	*P. sylvestris* (sw)	FT-MIR, KBr	Pandey and Pitman 2004
*Poria (Postia) placenta* (br)	*P. sylvestris* (sw)	^13^C CPMAS NMR	Sivonen et al. 2003
*C. subvermispora* (wr)	*P. abies* (sw)	FT-NIR (diffuse reflectance)	Schwanninger et al. 2004a
*C. puteana* (br)	*P. abies* (sw)	^13^C CPMAS NMR	Boonstra et al. 2006
*G. trabeum* (br)	*P. sylvestris* (sw)		
*P. placenta* (br)			
*C. puteana* (br)	*P. sylvestris*	^13^C CPMAS NMR	Irbe et al. 2006
*P. placenta* (br)			
Bjerkandera adusta, C. subvermispora*,	*P. abies* (sw)	FT-NIR(diffuse reflectance),	Fackler et al. 2006
*Daedaleopsis confragosa*, *Dichomitus squalens; Hypoxylon fragiforme*, *Oxyporus latemarginatus*, *Phlebia (Merulius) tremellosa(us)*, *Phlebia brevispora*, *Phlebia radiata*, *Pycnoporus sanguineus*, *Trametes cervina*, *Trametes pubescens*, T. versicolor*, )	*F. sylvatica* (hw)	FT-MIR, KBr*	
*Tyromyces chioneus* (wr			Fackler et al. 2007a
*C. puteana*, G. trabeum*, P. placenta* (br)			Fackler et al. 2007b
*Chaetomium globosum* (sr)			Fackler et al. 2007c
			Schmutzer et al. 2008
*Pycnoporus sanguineus* (wr)	*Pinus taeda* (sw)	^13^C CPMAS NMR	Levin et al. 2007
*T. versicolor* (wr)	*Pinus roxburghii* (sw)	FT-MIR, KBr	Pandey and Nagveni 2007
*T. hirsuta* (wr)	*Hevea brasiliensis* (hw)		
*Polyporus meliae* (br)			
*T. versicolor, G. applanatum* (wr)	*Cupressus glauca* (sw)	^13^C CPMAS NMR	Okino et al. 2008
*G. trabeum, L. lepideus* (br)			
*Laetiporus sulphureus* (br)	*Quercus alba* brown-rot lignin (hw)	^13^C CPMAS NMR	Koenig et al. 2010
*Coniophora puteana* (br)	*P. sylvestris* (sw)	DRIFT	Irbe et al. 2011
*P. placenta, G. trabeum*	*P. abies* (sw)	FT-NIR (transmission)	Fackler and Schwanninger 2010, 2011
*Physisporinus vitreus*	*P. abies* (sw)	UV microscopy	Lehringer et al. 2011

*br* brown-rot fungus, *wr* white-rot fungus, *sr* soft-rot fungus, *sw* softwood, *hw* hardwood

Provided that a higher number of degraded samples with a wider range of compositional differences were available, regression models to predict the chemical composition of degraded samples could be built. In particular, fast FT-IR methods can be used for this purpose. In mid infrared, univariate regression using ratios of bands has been often employed (Pandey and Pitman 2004). However, the relative intensity of reference bands may not be independent from alterations of the molecular structure (Barsberg 2010; Barsberg et al. 2011), and, thus, making use of the entire spectral information (multivariate calibration) is beneficial for regression models (Ferraz et al. 2000). Chemical properties such as lignin and polyose contents can be predicted from FT-MIR (Fackler et al. 2007b; Ferraz et al. 2000) and FT-NIR spectra (Fackler et al. 2007b; Schwanninger et al. 2004a) of fungal degraded samples as well as their physical properties: mass loss and density (Backa et al. 2001; Fackler et al. 2007b; Fackler et al. 2007c; Green et al. 2011; Kelley et al. 2002; Stirling et al. 2007; Sykacek et al. 2006) and the mechanical property compression strength (Green et al. 2011) could be analysed with NIR and MIR spectroscopy. Fackler et al. (2007a) connected mass loss and delignification, evaluated the selectivity of various strains of white-rot fungi on spruce and beech wood and found out that selectivity was more pronounced in the early phase when the mass loss was still low.

PCA of NIR spectra was useful to differentiate between the different decay types (Fackler et al. 2007c), different decay days (Schwanninger et al. 2004a), between brown-rot fungi of the order Boletales (*Coniophora puteana, Serpula lacrymans)* and *Gloeophyllum trabeum* (Gloeophyllales) and *Postia placenta* (Polyporales) (Jellison et al. 2002) and even between strains of the same species (Schwanninger et al. 2004a).

### Changes of wood chemistry

A number of early MIR studies are available that indicate oxidative modification of lignin during white-rot of wood. Kirk and Chang (Kirk 1975; Kirk and Chang 1975) found that carboxyl groups were formed on spruce wood lignin by both the white-rot fungi *Coriolus versicolor* (*Trametes versicolor*) and *Polyporus anceps* and by the brown-rot fungus *Lenzites trabea* (*G. trabeum*) and that the bulk of these carboxyl groups was conjugated to aromatic rings. Faix et al. (1991) reported similar results from white-rot (*T. versicolor, Pleurotus ostreatus* and *Lentinula edodes*)-degraded beech wood. Increase of a C═O band was described as indicative for incipient brown-rot of pine wood (Körner et al. 1990).

**Fig. 1:**
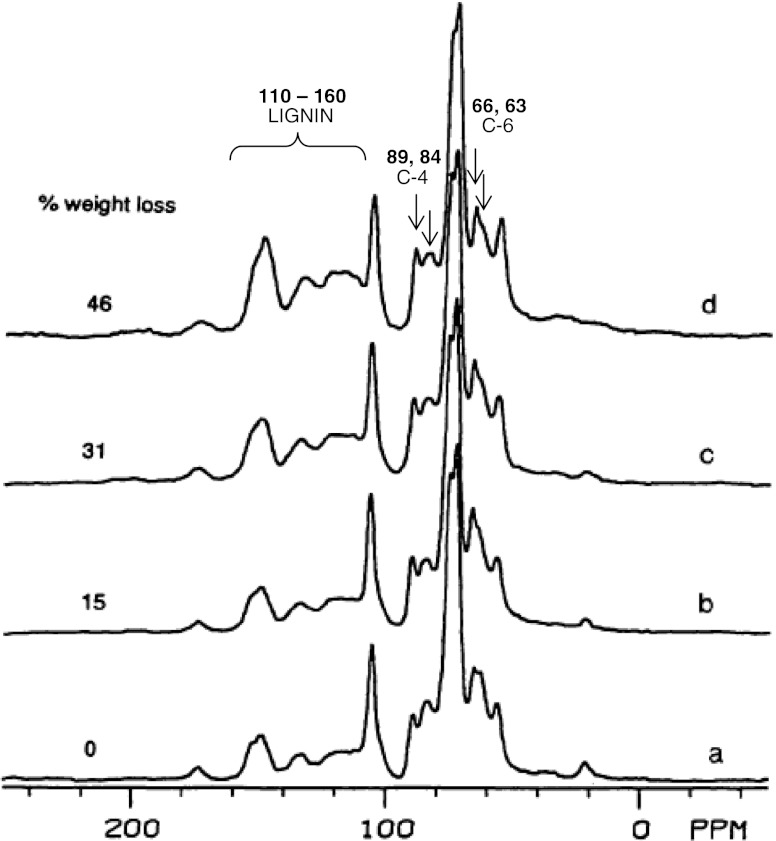
The ^13^C CPMAS NMR spectra show the preferential removal of amorphous carbohydrates (63 and 84 ppm) relative to crystalline cellulose (66 and 89 ppm) and accumulation of aromatic lignin structures (110–160 ppm) and a changed pattern of the respective region indicating lignin modification after brown-rot of Colorado blue spruce wood caused by *P. placenta.* Reproduced from Davis et al. (1994c) with permission from de Gruyter

Davis et al. (1994a, b, c) (Fig. 1) studied white-rot (*P. chrysosporium, T. versicolor* and *Dichomitus squalens*) and brown-rot (*P. placenta*) decay of a softwood (Colorado blue spruce, *Picea pungens*) and a hardwood (paper birch, *Betula papyrifera*) with ^13^C CPMAS NMR. Their results indicated a small preference for the degradation of amorphous polysaccharides compared to crystalline cellulose by white-rot fungi, which was even smaller on beech wood. They also reported a decrease of the syringyl (S) to guaiacyl (G) ratio of lignin as decay proceeded and suggested a direction of decay from the lumen towards the cell wall rather than the preference of the fungi for S structures over G structures, because in hardwoods, S structures are enriched in secondary cell wall layers, whereas the content of G structures is higher in middle lamellae and outer cell wall layers (Fengel and Wegener 1989). Major evidence for oxidative C_α_-C_β_ cleavage caused by white-rot fungi was found only in softwoods, but the authors did not rule out the possibility that oxidised structures were just metabolised more easily in hardwoods than in softwoods and therefore were not present in the degraded wood. The brown-rot fungus showed a clear preference for amorphous polysaccharides, with crystalline cellulose being accumulated as decay proceeded. This preference was smaller however on birch wood than on spruce wood. Softwood lignin lost methoxyl groups during brown-rot, and vanillic acid type structures were formed. Significant ring cleavage was not found. They also reported cleavage of β-*O*-4 linkages by the brown-rot fungus as the main reaction of the hardwood lignin structures. This mechanism of lignin cleavage of spruce wood by the brown-rot fungus *G. trabeum* was confirmed later with HSQC-solution state NMR spectroscopy (Yelle et al. 2008). Fackler and Schwanninger (2010) compared methyl ether and aryl ether cleavage by the polyporaceae *G. trabeum* and *P. placenta* by means of polarisation FT-NIR spectroscopy in samples of up to 16 % mass loss and concluded from the lack of relative orientation of the formed phenols that both routes to take place to similar extents. Yelle et al. (2008; 2011) related benzaldehydes, benzoic acids and arylglycerols to methoxyl in sound and brown-rot degraded spruce (*Picea abies*, 64 % mass loss) and aspen wood (*Populus* sp., 70 % mass loss). They also found that a strong preference for aryl ether cleavage in the softwood and only a slight preference on the hardwood at these advanced stages.

In their imaging IR spectroscopy studies, Fackler et al. (2010, 2011) found a significant reduction of glycosidic linkages within polysaccharides in relation to carbohydrate ring structures in brown-rot (*G. trabeum, P. placenta*)- and in white-rot (*T. versicolor*)-degraded wood. These findings were highly indicative for incipient brown-rot and early white-rot and corroborated in a non-destructive way early findings by Cowling (1961) who found an increase of extractable polysaccharide degradation products in brown-rot wood.

### Wood molecular structure

A preference for amorphous polysaccharides over crystalline cellulose is well known for many decay fungi. A closer look into the molecular structure of the degraded wood cell walls was provided by solid state NMR and by infrared methods.

Gilardi et al. (1994) studied white-rot (*T. versicolor*)- and brown-rot (*C. puteana*)-degraded pine and beech wood with wide-line solid state NMR. They found increased mobility of the molecular components of the cell wall in the degraded wood. Kim and Newman (1995) found an overall reduced surface to interior ratio of crystalline cellulose in brown-rot (*G. trabeum*)-degraded softwood wood (*Pinus koraiensis*) and concluded that crystallites of narrower dimensions—cellulose I_α_ crystallites located in middle lamellae and primary cell walls (Kataoka and Kondo 1998)—were degraded in preference to cellulose I_β_ crystallites of secondary cell walls.

**Fig. 2:**
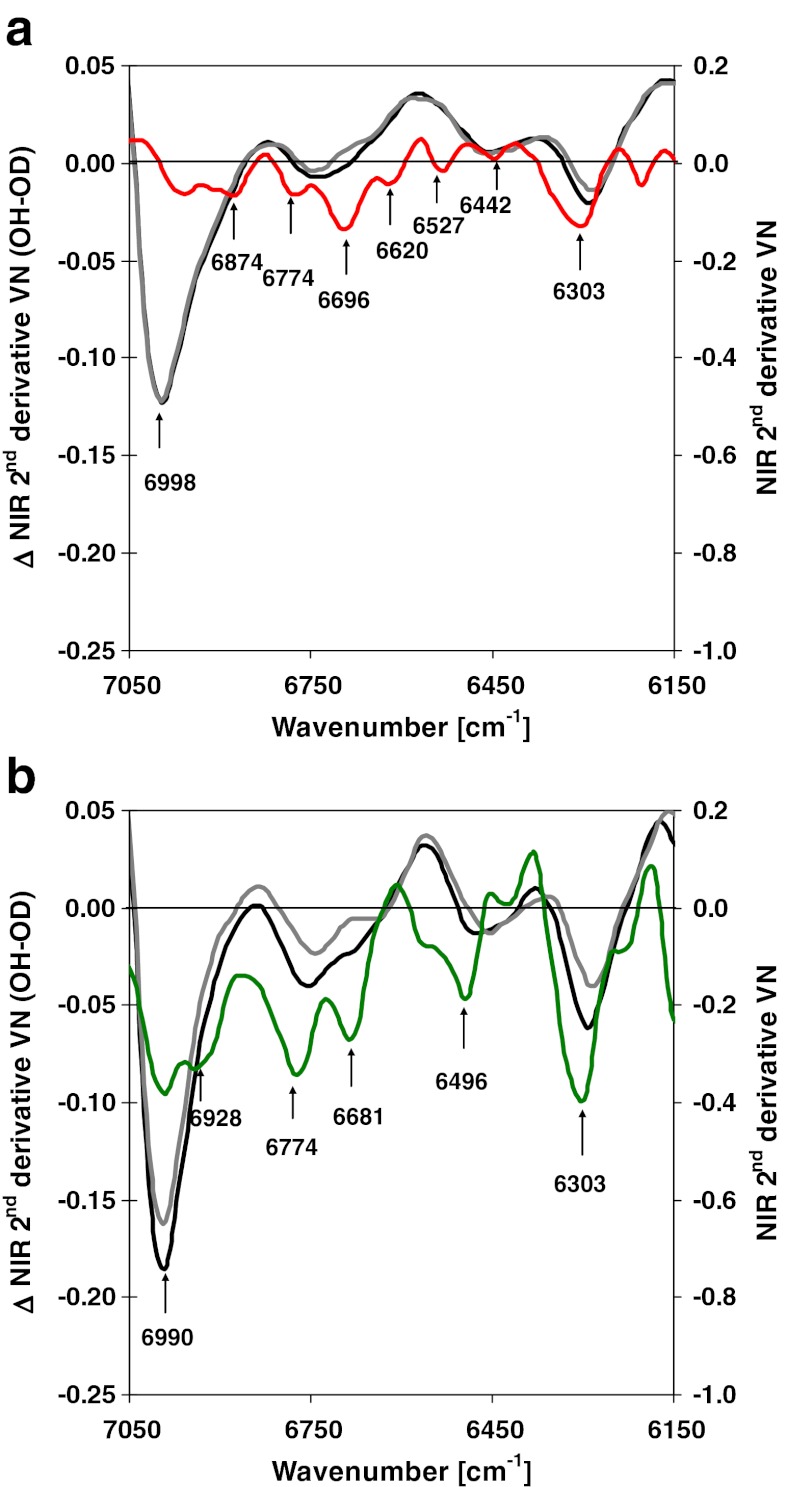
Second derivatives of FT-NIR transmission spectra of spruce wood in the OH first overtone region assigned to alcoholic and phenolic OH groups (6,928 cm^-1^) before (*black line*) and after (*grey line*) impregnation with deuterium oxide and differences (*Δ NIR*) between them (*coloured line*) demonstrate the accessibility of OH groups to deuterium oxide. The derivatives of the NIR spectra were vector normalised (*VN*) and show the lower number of OH groups due to polysaccharides degradation and a reduced accessibility of these groups in brown-rot wood with 16 % mass loss (**a**) compared to non-degraded spruce wood (**b**) Reproduced after Fackler and Schwanninger (2011) with permission from IM publications

Popescu et al. (2010a) reported the apparent increase of crystallite size in lime (*Tilia cordata*) wood degraded by *Trichoderma viride*—a wood-inhabiting deuteromycete—and assigned this effect to higher mobility of less ordered polysaccharide chains after selective degradation of amorphous polysaccharides. The authors investigated the same degraded wood with 2D-correlation FT-MIR spectroscopy and found decreased carbohydrate content and decreased crystallinity. Selective degradation of the amorphous fraction (glucomannan and amorphous cellulose) was found for brown-rot (*G. trabeum* and *P. placenta*)-degraded spruce wood by means of polarised FT-NIR spectroscopy (Fackler and Schwanninger 2010). In a later study employing polarised FT-NIR in combination with deuterium exchange, the same authors (Fackler and Schwanninger 2011) found accessibility and hydrophilicity loss in dried brown-rot wood even before a substantial mass loss occurred (Fig. 2). This loss was most pronounced at amorphous polysaccharides in middle lamellae, primary cell walls and outer secondary cell walls and indicated structural changes within these cell wall regions during the incipient stage of degradation.

Alterations of interactions of white-rot (*Ceriporiopsis subvermispora*) wood with water, namely increased hydrophilicity, was reported by Ferraz et al. (2004) and by Schwanninger et al. (2004a). Both groups employed NIR spectroscopy. The first group of authors reported that dried biodegraded wood absorbed water more rapidly than the non-decayed control; the second group reported more water adsorbed to cellulose even after thoroughly drying the milled samples and found significant differences between three strains.

### Localisation of degradation

In the previous chapter, it was shown that some conclusions on the localisation of incipient and early wood degradation processes can be drawn without employing microscopy techniques, when the anisotropic nature of wood and the orientation and molecular structure of its macromolecular constituents in its cell wall layers are considered.

**Fig. 3:**
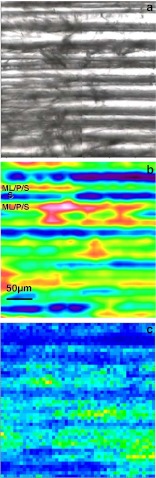
Localisation of early brown-rot degradation within a radial thin section of spruce wood with FT-IR imaging microscopy: **a** CCD camera image of 10 tracheids of a degraded spruce wood section from a sample degraded by the brown-rot fungus *G. trabeum* for 4 weeks. **b** FT-IR pseudo-colour spectral absorbance image. Zones of high total absorbance (*red*) show a high contribution of middle lamella (*ML*) and primary cell walls (*P*) and outer layers of secondary cell walls (*S*1). Those with low total absorbance (*blue*) can be assigned to regions with a high contribution of secondary cell walls (*S*2) and pits. **c** Example of a partial least squares discriminant analysis image gained through multivariate image analysis—degraded pixels are plotted *yellow to light green* and are found in ML/P/S1 regions of the section in 6.25 μm pixel resolution

Microspectroscopic techniques, however, provide direct access to spatially resolved information, and IR imaging microscopy allowed for localisation of incipient and early decay processes in white-rot (*T. versicolor* and *C. subvermispora*)- and brown-rot (*G. trabeum* and *P. placenta*)-degraded spruce earlywood tracheids (Fackler et al. 2010, 2011) (Fig. 3). Incipient brown-rot processes could be localised in outer cell wall regions allocated to middle lamellae, primary cell walls and outer secondary cell walls, whereas white-rot processes were evenly distributed in the tracheid cells, even if the cells in one thin section were affected to very different extents. Indicative for incipient brown-rot processes was the reduction of glycosidic linkages when sugar moieties had not yet disappeared from wood. Cleavage of polysaccharides prior metabolisation of carbohydrates was to some minor extent also found during white-rot by *T. versicolor* and was not significant during selective white-rot by *C. subvermispora*.

Due to its much higher spatial resolution (~250 nm), UV microspectroscopy allows for investigating degradation processes on the subcellular level. Bauch et al. (1976) investigated changes of the lignin content of wood cell walls during white-rot, brown-rot and soft-rot (Table 2) and found a decrease of the lignin content in soft-rot-degraded secondary walls of hardwood fibres in vicinity to cavities.

Kleist and Schmitt (2001) studied brown-rot degradation of Sapelli wood and found that at high moisture content, *C. puteana* caused a soft-rot type of decay with rhomboidal cavities in secondary cell wall, but no differences in lignin content of adjacent cell wall regions could be evidenced by UV microscopy.

**Fig. 4:**
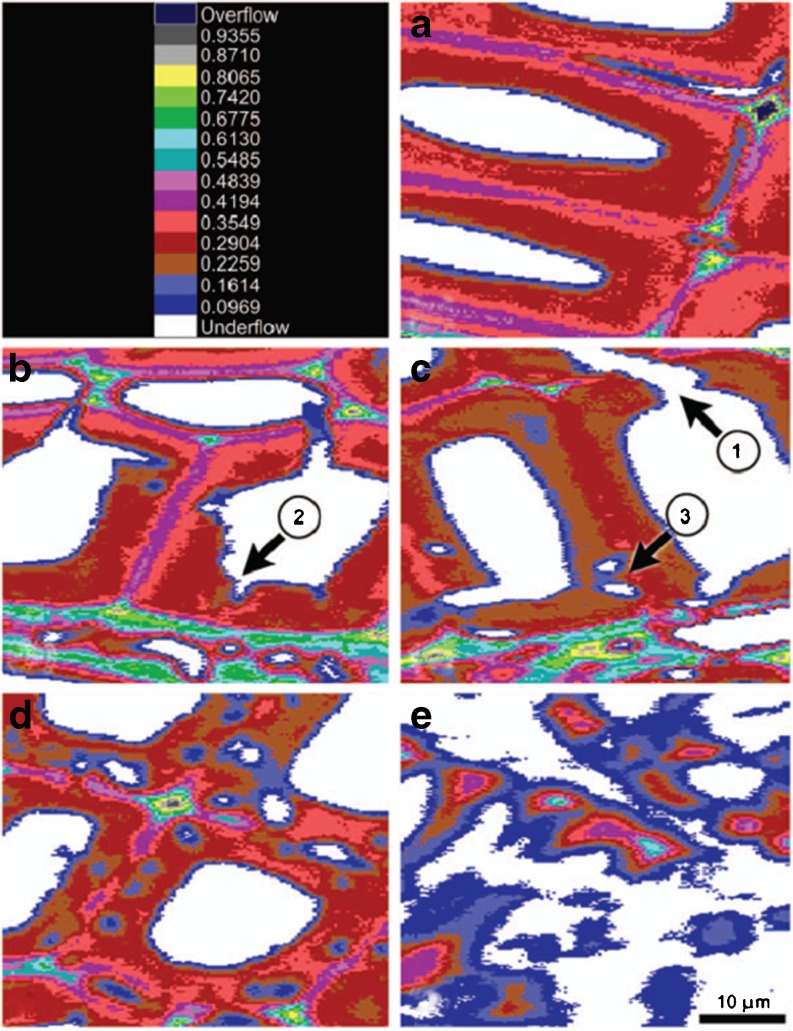
UV microscopic images of Norway spruce latewood after zero (**a**), 7 (**b**), 9 (**c**), 12 (**d**) and 32(**e**) weeks of incubation with the white-rot fungus *P. vitreus*. The colour pixels represent different UV_280_ absorbances. Hyphal tunnelling (*1*), notches (*2*) and cavities (*3*) can be observed as proof for simultaneous degradation. The progress of selective delignification is visible by lowered absorbances in areas of the secondary cell wall layers. Reproduced from Lehringer et al. (2011) with permission from de Gruyter

Modern UV imaging systems allow for a clear visualisation of the wood composition that is derived from the aromatic absorbance band due to lignin (Fig. 4). Very recently, Lehringer et al. (2011) reported two types of degradation patterns, when *Physisporinus vitreus*, commonly described as a selective white-rot fungus, degraded spruce wood: selective delignification leading to reduced lignin content of cell walls and a soft-rot type of decay characterised by hyphal tunnelling with bore holes surrounded by cell wall regions with reduced lignin content.

## Conclusions and outlook

Nuclear magnetic resonance, IR and UV spectroscopy provide valuable and less invasive or even non-destructive tools to study wood chemistry, physics and molecular structure in various stages of softwood and hardwood degradation by white-rot, brown-rot and soft-rot fungi. The methods reviewed here allow for the analysis and characterisation of fungal degradation processes on their actual substrate wood and do not rely on model systems of isolated wood constituents or synthesised model compounds. Some methods have been proven to be sensitive enough to detect and characterise early or even incipient stages of decay.

The detection of compositional changes in degraded wood, however, is due to high variability of the raw material and due to uneven degradation processes often restricted to degradation stages with significant mass loss and changes of the visual appearance of the substrate. Thus, many studies reviewed here were carried out with strongly degraded wood.

Microspectroscopy methods allow for studying extreme situations in unevenly degraded samples and therefore are applicable for detecting decay processes very early. To assess the significance of differences caused by fungal decay and resolve them from natural variations found in non-decayed wood, sound data and image analysis techniques are required; otherwise, early decay may remain undetected. Of the microspectroscopy methods, UV microspectroscopy provides the highest spatial resolution, whereas the advantage of infrared microspectroscopy is the higher information content.

Both NMR and MIR spectroscopy confirmed molecular mechanisms involving one-electron oxidants and consecutive formation of α-carbonyls for many species of white-rot fungi on a number of different wood species of both softwoods and hardwoods. One-electron oxidation, putatively by free hydroxyl radicals, also plays a significant role in lignin degradation by brown-rot fungi, during which methoxyl and aryl ether structures are cleaved, and oxidised structures are formed.

Among the wood polysaccharides, amorphous structures are degraded in preference to crystalline ones, albeit this preference depends on the species of the fungus and on the wood species. The accumulation of polysaccharide degradation products of lower molecular mass during early brown-rot preceding their uptake and respiration by the fungi has been suggested or demonstrated with non-destructive means independently with wide-line NMR spectroscopy and FT-IR imaging microscopy.

Infrared imaging microscopy allowed for the localisation of incipient brown-rot degradation processes in outer cell wall regions (middle lamellae, primary or outer secondary walls). These findings corroborated earlier NMR studies carried out under consideration of the molecular structure of wood cell walls: brown-rot fungi were found to degrade smaller cellulose I_α_ crystallites of primary cell walls and middle lamellae in preference to larger cellulose I_β_ crystallites of secondary cell walls, and with polarised FT-NIR spectroscopy, it was found that less ordered and transversely aligned polysaccharides of outer cell wall regions were modified in preference to longitudinally aligned ones of secondary cell walls. In contrast to brown-rot, white-rot has been shown to be distributed quite evenly in softwood tracheids when investigated with IR microscopy; with UV microscopy, with its higher spatial resolution, it was possible to detect different morphologies and different extents of delignification within the cell walls.

The most sensitive markers for the detection of incipient decay assessed by the spectroscopic methods reviewed here were the depolymerisation of wood polysaccharides (Fackler et al. 2010) reflected as the loss of the IR band of the glycosidic bond of polysaccharides, and loss of accessibility of distinct OH groups in dried degraded wood (Fackler and Schwanninger 2011). It may be speculated that also wide-line NMR would be applicable to detect much smaller differences than shown by Gilardi et al. (1994), because differences of mobility of cell wall components and water were highly significant between the investigated specimens in advanced degradation stages.

### Future potential of the methods

Nuclear magnetic resonance, IR, and UV spectroscopy proved to be successful and highly significant to study wood properties related to intermediate and advanced decay. Although it has been shown that the characteristics of fungal wood degradation may change during decay (Cowling 1961; Fackler et al. 2007a), particularly the NMR methods, unfortunately, have been scarcely employed to study incipient and early degradation processes. IR spectroscopy on the contrary had been challenged to its limitations with statistic means because the short data acquisition times characteristic for IR spectroscopy are well suited for multivariate data analysis of high numbers of spectra. IR spectroscopy has also a great potential for in situ studies of decay processes.

UV microscopy offers the highest spatial resolution and has got a high potential for further investigations of incipient and early decay processes in distinct wood cell wall layers of various cell types.

For future studies, it will be advantageous to combine knowledge on the alterations of wood chemistry and structure during decay gained through spectroscopic and microspectroscopic methods with that on biochemical processes taking place while wood is degraded by the fungi. This approach calls for interdisciplinarity to be able to connect spectroscopy to ultrastructural and immunochemical analyses with e.g. electron microscopy, X-ray diffraction or atomic force microscopy as well as to biochemistry and molecular biology.

Within the Basidiomycota, species of wood decay fungi are found in many orders, and comparative genomic analyses of 31 fungal genomes revealed that brown-rot fungi evolved at least four times from white-rot ancestors and therefore may differ from each other (Floudas et al. 2012). Spectroscopic studies of degraded wood, however, by now, rarely accounted for biological systematics within the wood-degrading Agirocomycetes but rather focussed on the decay type. Often, aggressive strains and wood species recommended for standardised decay tests have been investigated (e.g. European standard EN 113); other studies were related to industrial applications of white-rot fungi. More systematic comparative spectroscopic studies on the same substrate considering fungal phylogenies would be desirable to be able to find differences particularly among the brown-rot fungi.

Apparent slow and asynchronous growth of hyphae in lignocellulosic substrates leads to uneven decay caused by brown-rot and by white-rot fungi even in adjacent wood cells (Fackler et al. 2010, 2011) and different decay morphology—selective and simultaneous white-rot caused by *Heterobasidium annosum*—in the same piece of wood was reported very early by Hartig (1878) and demonstrated with UV microscopy by Lehringer et al. (2011). Experimental tools to identify the metabolic state of a hypha therefore would be required. The combination of spatially resolved biochemical data with spatially resolved microspectroscopy data could bring this research field further step forward.

## Footnotes

1 Analysis: greek “ανάλυση – *analyse”,* breaking up
